# Nano-engineering the material structure of preferentially oriented nano-graphitic carbon for making high-performance electrochemical micro-sensors

**DOI:** 10.1038/s41598-020-66408-9

**Published:** 2020-06-10

**Authors:** Edoardo Cuniberto, Abdullah Alharbi, Ting Wu, Zhujun Huang, Kasra Sardashti, Kae-Dyi You, Kim Kisslinger, Takashi Taniguchi, Kenji Watanabe, Roozbeh Kiani, Davood Shahrjerdi

**Affiliations:** 1 Electrical and Computer Engineering, New York University, Brooklyn, NY 11201 USA; 2 National Center for Nanotechnology and Semiconductors, KACST, Riyadh, 11442 Saudi Arabia; 3 Center for Neural Science, New York University, New York, NY 10003 USA; 4 Center for Quantum Phenomena, Physics Department, New York University, New York, NY 10003 USA; 5 Center for Functional Nanomaterials, Brookhaven National Laboratory, Upton, NY 11973 USA; 6 National Institute of Materials Science, 1-1 Namiki Tsukuba, Ibaraki, 305-0044 Japan; 7 Department of Psychology, New York University, New York, NY 10003 USA

**Keywords:** Materials for devices, Graphene, Synthesis and processing, Synthesis of graphene

## Abstract

Direct synthesis of thin-film carbon nanomaterials on oxide-coated silicon substrates provides a viable pathway for building a dense array of miniaturized (micron-scale) electrochemical sensors with high performance. However, material synthesis generally involves many parameters, making material engineering based on trial and error highly inefficient. Here, we report a two-pronged strategy for producing engineered thin-film carbon nanomaterials that have a nano-graphitic structure. First, we introduce a variant of the metal-induced graphitization technique that generates micron-scale islands of nano-graphitic carbon materials directly on oxide-coated silicon substrates. A novel feature of our material synthesis is that, through substrate engineering, the orientation of graphitic planes within the film aligns preferentially with the silicon substrate. This feature allows us to use the Raman spectroscopy for quantifying structural properties of the sensor surface, where the electrochemical processes occur. Second, we find phenomenological models for predicting the amplitudes of the redox current and the sensor capacitance from the material structure, quantified by Raman. Our results indicate that the key to achieving high-performance micro-sensors from nano-graphitic carbon is to increase both the density of point defects and the size of the graphitic crystallites. Our study offers a viable strategy for building planar electrochemical micro-sensors with high-performance.

## Introduction

Carbon materials are widely used in building electrochemical sensors for detecting biomolecules because of their favorable electrochemical activity, bio-compatibility, rich surface chemistry, and strong resistance to bio-fouling. In biomolecule sensing applications, it is desirable to implement a large-scale sensing system comprising many small (micron-sized) carbon electrodes with high packing density. However, such large-scale systems are challenging to implement. In particular, existing implementations are limited mainly to one or a handful of carbon electrodes^1–5^.

Significant progress has been made on the development of single-electrode micro-sensors from bulk carbon materials, such as carbon fibers^6,7^ and nanotube yarns^8,9^. However, the large cylindrical form of these materials (>5 μm diameter) limits them to the fabrication of single or small-array micro-sensors. Importantly, while past research on this topic has evaluated a wide variety of carbon-based materials for boosting the sensor performance, the search for an optimal carbon material is still ongoing^10–13^. It is generally accepted that, in a carbon material, defects and functional groups influence the sensitivity and the charging current of carbon-based electrochemical sensors. However, from a fundamental standpoint, a detailed understanding of the underlying electrochemical mechanisms that control these sensor characteristics, i.e. electron transfer rate and electrode capacitance, is still a subject of research^14–17^.

Due to the above-mentioned limitations of bulk carbon materials in producing a dense sensor array, one promising strategy is to form thin-film carbon materials on dielectric substrates using standard microfabrication techniques. This generally involves converting lithographically-defined polymeric islands into pure sp^2^ hybridized carbon through a high-temperature thermal treatment (a process known as pyrolysis)^18–25^. Of various substrates, dielectric-coated silicon is an attractive choice because of its low cost, its commercial availability in large dimensions (up to 300 mm diameter), and its compatibility with standard micro-fabrication techniques. The latter feature is particularly useful for making functional sensor carriers from silicon substrates, e.g., by shaping silicon into narrow and flexible shafts for applications in neural interfacing. However, the thermal stability of silicon substrates limits the production temperature of these microfabricated thin-film carbon materials to below 1100 °C. Due to this temperature limit, the resulting material has a fully disordered sp^2^ structure with slow electron transfer kinetics. Hence, even though this microfabrication process provides a simple method for making thin-film pyrolyzed carbon materials, the resulting sensors have poor sensing characteristics^18,26^.

A possible solution is to ensure that the resulting thin-film carbon materials have graphitic structures. Graphitic structures are favorable for realizing highly sensitive electrochemical sensors due to their inherently fast electron transfer kinetics. This can be achieved by adding metal catalysts, such as nickel (Ni), which are known to promote the transformation of solid-state amorphous carbon to graphene^27^. Performing pyrolysis in the presence of metal catalysts is a known technique, often referred to as metal-induced graphitization. The primary focus of the early research on this material synthesis technique, however, has been on the production of bulk sp^2^ hybridized carbon materials mostly for applications in energy storage^28–30^. A new direction, more relevant for building miniaturized sensors, has been to apply this technique for making mono- and few-layer graphene on dielectric-coated substrates^31–37^. The main focus of these studies has been to generate defect-free graphene materials, which are ideal for high-speed electronic switches, but not for electrochemical sensors, where the performance strongly depends on structural defects^17,26,38–47^. However, the application of metal-induced graphitization for making thin-film sensing materials remains under-developed. It is generally unclear how to optimize parameters of the synthesis process, and hence nano-engineer the ensuing material structure for achieving desirable electrochemical performance.

In particular, a large number of parameters could influence the material structure during the synthesis process, hence trial-and-error approaches are inefficient for finding the right parameter settings that result in a favorable sensor performance. Optimization of the parameters could be performed efficiently if one has access to quantitative phenomenological models that relate the main characteristics of the sensor to quantifiable and tunable properties of the material structure. Such models can guide the material engineering by precisely tuning the most relevant structural properties, eliminating the need for trial-and-error approaches.

In a previous study^26^, we reported on quantitative principles for engineering the area-normalized sensitivity of graphene-based sensors for fast-scan cyclic voltammetry (FSCV) measurements of dopamine. While increasing the sensitivity (the amplitude of redox current) per unit area is crucial for building compact sensors, and hence enhancing the spatial resolution, optimizing this sensor characteristic alone is inadequate for improving the detection limit. In particular, besides the redox current (which is the signal of interest), the FSCV measurements generate a background charging current, which in general has a significantly larger amplitude than the redox current. The existence of this current in sensing experiments negatively impacts the detection limit, primarily due to the strong constraints that it imposes on the quantization error of the readout electrical circuit^48^. To improve the limit of detection, it is hence crucial to increase the ratio of the redox current amplitude (i.e., signal) to the background current amplitude. We refer to this metric as the S-B ratio.

Here, we build on our previous study and report a material nano-engineering strategy for making high-performance electrochemical micro-sensors with a superior S-B ratio. First, we introduce a variant of metal-induced graphitization for producing preferentially oriented nano-graphitic (NG) carbon materials, which are graphitic films with nanoscale crystallite size. A new feature of our synthesis method is that, through substrate engineering, the orientation of graphitic planes within the film preferentially aligns with the silicon growth substrate. As we explain later, this feature allows us to use Raman spectroscopy to quantify the structural properties on the sensor surface where the electrochemical processes occur. Then, we study the relationship between the FSCV characteristics of the micro-sensors and the structural properties of the NG carbon materials. We develop phenomenological models for predicting the amplitudes of the redox current and the capacitance, normalized to the sensor area, based on structural defect densities in the material. We show that the combination of these two models is a good predictor for the S-B ratio. Our results indicate that, for the range of the defect densities studied here, the S-B ratio may be optimized by increasing both the density of point defects and the size of the graphitic crystallites in NG carbon materials. This study provides a simple and efficient material engineering strategy for the fabrication of miniaturized sensors with superior electrochemical properties.

## Results

### Synthesis of NG carbon material

**Figure 1:**
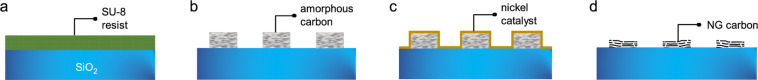
Synthesis of micron-scale NG carbon islands. Schematic illustration of the main steps: (**a**) applying SU-8 resist onto SiO_2_, (**b**) creating micron-scale amorphous carbon islands through e-beam lithography and carbonization, (**c**) depositing Ni catalyst under UHV conditions, (**d**) transforming amorphous carbon islands into NG carbon islands through graphitization at 1100 °C.

The illustrations in Fig. 1 show the main steps of our synthesis method. An advantageous feature of our method is the production of small micron-scale NG carbon islands from pre-patterned amorphous carbon directly on thermally grown SiO_2_ substrates. This direct growth technique significantly simplifies the sensor fabrication process because it avoids the common challenges associated with the graphene transfer techniques such as the formation of cracks^49,50^, wrinkles^51^, or the chemical modification of graphene due to contamination^52^. To produce the amorphous carbon islands, we first used electron-beam lithography (EBL) to pattern the SU-8 resist into islands with desired shapes and dimensions.

We then performed a carbonization step by annealing the SU-8 islands at 450 °C in a non-oxidizing ambient. Past studies have established that this carbonization process converts the polymeric films into an amorphous sp^2^ hybridized carbon^53–55^. We then performed the solid-state transformation of the amorphous carbon into an NG carbon material through metal-induced graphitization. This process involves two main steps. The first step is the deposition of an ultra- thin (sub-2 nm) Ni as the metal catalyst in ultra-high vacuum (UHV) environment. The second step is the annealing of the samples at 1100 °C.

Unlike the past studies^31–37^ that aimed at producing defect-free graphene by using a thick metal catalyst (> 50 nm), our objective is to produce films containing specific structural defects because of the critical effect of defects on the electrochemical properties of carbon materials^17,26,38–47^. In our experiments, we observed that the thickness of the metal catalyst had a noticeable effect on the density of structural defects in the resulting NG carbon material. Hence, to study the correlation between the density of defects and the sensor characteristics, we produced multiple samples by varying only the thickness of the metal catalysts while keeping other processing conditions unchanged. Those processing conditions include the graphitization temperature, the background carbon vapor pressure, the hydrogen flow rate, and the metal deposition rate. To avoid unintentional incorporation of impurities, we used ultra-high purity Ni (99.999%) catalysts and carrier gases and performed metal depositions in a UHV environment. This experimental design allowed us to isolate the effect of the density of structural defects by controlling other factors that might influence the electrochemical properties of the sensors.

**Figure 2:**
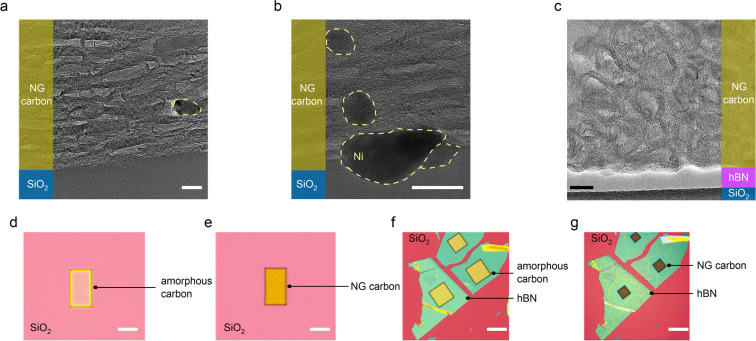
Preferential directionality of graphitic planes. (**a,b**) Cross-sectional TEM images of an NG carbon film directly on SiO_2_ at two different magnifications. The Ni clusters inside the NG carbon film are marked with dashed yellow lines. (**c**) Cross-sectional TEM image of an NG carbon film on hBN, showing ring-like graphitic planes with random orientations within the film. Scale bars in TEM images are 20 nm. Example optical images of amorphous carbon islands on (**d**) SiO_2_ and (**f**) hBN. The optical images of the same islands after graphitization on (**e**) SiO_2_ and (**g**) hBN. Scale bars in optical images are 30 µm.

In Fig. 2(a,b), we show two examples of the cross-sectional transmission electron microscopy (TEM) images of an NG carbon film grown directly on SiO_2_. In our TEM studies, we observed the segregation of Ni into nanoscale particles inside the NG film, predominantly residing at or near the interface with the SiO_2_ layer. This observation is consistent with a recent study on the growth of graphene films using metal-induced graphitization technique, reporting the rapid diffusion of Ni catalyst to the bottom of the carbon film during the graphitization process^31^.

The TEM studies also revealed a new and striking feature of our synthesis process: a preferential (but not perfect) directionality of the graphitic crystallites within the film, which grew parallel to the substrate (see Fig. 2(a,b)). We achieved this feature by applying an in-plane tensile strain to the amorphous carbon islands during the heat treatment. This strategy builds on the past reports on the production of pyrolytic graphite (HOPG)^56,57^. Those past studies found that stretching free-standing carbon films during the heat treatment results in films with highly oriented graphitic planes, whereas, in the absence of the mechanical pulling, the basal planes grow at random orientations. In our experiments, we developed a substrate engineering approach for applying an in-plane tensile strain to the carbon islands. Our substrate engineering approach relies on two factors: the adhesion of carbon islands to the substrate, and the mismatch in the coefficient of thermal expansion (CTE) between the carbon islands and the substrate. The combination of these two factors causes the carbon islands to experience an in-plane tensile strain during heat treatment due to the expansion of the substrate. Based on the CTEs of silicon (∼4.5 × 10^−6^ K^−1^, ref. ^58^) and graphite (<1 × 10^−6^ K^−1^, ref. ^59,60^), we estimate a tensile strain of ∼0.4% during heat treatment at 1100 °C^61^. Note that the NG material resembles a highly defective HOPG, which has a lower CTE compared to defect-free graphite^59^, which could result in a higher level of tensile strain in the NG film during annealing. Using substrates with a higher CTE mismatch should enhance the strain.

The efficient application of the tensile strain requires a strong adhesion between the carbon islands and the growth substrate. The choice of the dielectric below the carbon islands plays a fundamental role in satisfying this requirement. Specifically, we hypothesized that the carbon islands could anchor more strongly to an imperfect dielectric material with unsatisfied surface bonds (i.e., dangling bonds) than to a perfect dielectric material with fully satisfied surface bonds. To study the effect of substrate choice, we produced carbon islands on two dielectric materials. For an imperfect dielectric, we used thermally grown SiO_2_, which is an amorphous material. For a perfect dielectric, we used exfoliated hexagonal boron nitride (hBN), which is a two-dimensional layered material^62^. Interestingly, but not surprisingly, we found that the perfect surface properties of exfoliated hBN allow the carbon islands to shrink freely (∼70% reduction in size) during heat treatment, as shown in Fig. 2(f,g). This property resulted in the growth of ring-like graphitic planes with random orientation within the film, as shown in Fig. 2(c). In contrast, the size of carbon islands on SiO_2_ reduces only slightly (∼5% reduction in size) during the heat treatment, as shown in Fig. 2(d,e). This observation suggests the strong adhesion of the carbon islands to SiO_2_ caused by the dangling bonds of the dielectric surface. These features of SiO_2_ enable the efficient transfer of the in-plane shear strain to the carbon islands, resulting in the preferential directionality of the graphitic planes (Fig. 2(a,b)).

**Figure 3:**
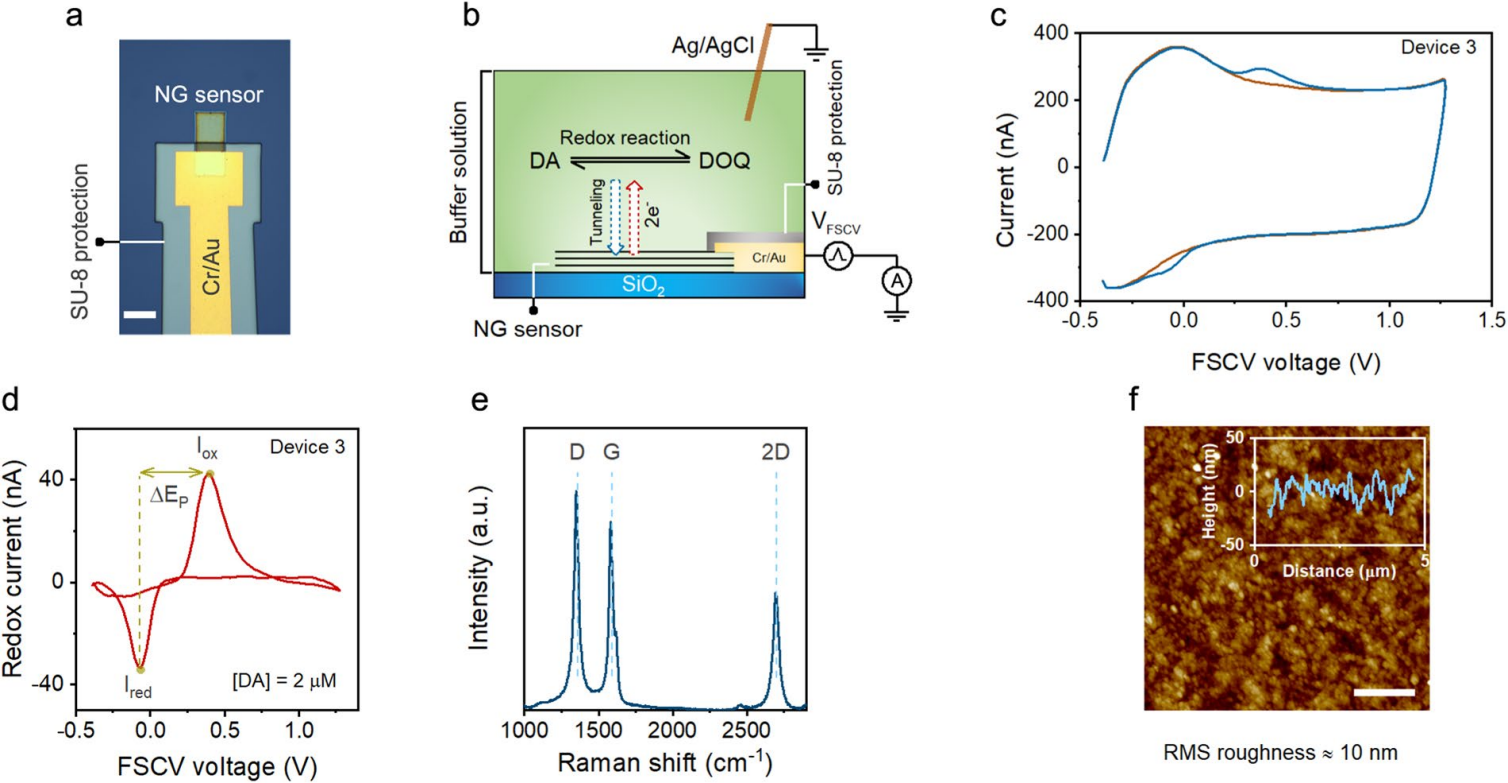
Characterization of NG micro-sensors. (**a**) Optical image of an example NG micro-sensor. Scale bar is 20 μm. (**b**) Cross-sectional schematic of our experimental setup for FSCV measurements of dopamine. A rapid potential sweep (V_FSCV_) was applied to the NG micro-sensor, and (**c**) the total current was measured with and without dopamine in a 1X PBS solution. (**d**) The subtraction of the measured currents before and after dopamine gives the cyclic voltammogram. Additional data showing the consistency of our NG micro-sensors are shown in Section S1 of Supplementary Information. (**e**) An example Raman spectrum of an NG micro-sensor. We used the Raman spectra of our sensors to quantify the average density of point defects and the size of the graphitic crystallites. (**f**) An example topographic image of an NG micro-sensor obtained using atomic force microscopy (AFM), showing an RMS roughness of ∼10 nm. The inset shows a line scan. Scale bar is 1 μm.

The methods we employ below to develop the phenomenological models for our micro-sensors take advantage of the preferential directionality of the graphitic planes. Note that because of the predominantly parallel orientation of the basal planes to the surface of our sensing material— where the electrochemical processes occur—sensor characteristics are determined by the structural properties of those basal planes. This property enables us to use non-destructive Raman spectroscopy to estimate the density of structural defects of our NG sensors as Raman spectroscopy provides information about the density of defects within the basal planes^63,64^. After synthesizing NG carbon materials containing different amounts of structural defects, we used the materials to produce micro-sensors employing standard micro-fabrication techniques. To do so, we first used a combination of EBL, e-beam evaporation, and lift-off to form low-resistance Cr/Au metal contacts to the sensors. We then patterned a SU-8 resist using EBL, which accurately defines the sensor area and protects the metal leads from coming into contact with the solution during *in vitro* experiments (see below). Figure 3(a) shows the optical image of an example miniaturized NG sensor.

### Electrochemical characterization of NG micro-sensors

Figure 3(b) shows the schematic illustration of the measurement setup. We studied the characteristics of our NG micro-sensors using the FSCV technique by applying rapid potential sweeps to the sensor and concurrently measuring the resulting current. We focused our study on dopamine (DA) as the target analyte primarily due to our interest in exploring the application of our miniaturized electrodes in future studies involving tonic (slow) and phasic (fast) dopamine release in the brain. In our experiments, we adopted a triangular voltage waveform, which previous studies have used extensively for FSCV measurements of dopamine^6,65^. Specifically, the waveform had a voltage range from −0.4 V to 1.3 V, a ramp rate of 400 V/s, and a repetition rate of 10 Hz. Figure 3(c) shows an example FSCV measurement. In the absence of dopamine, the total current consists only of the background current (I_bg_), which arises from the charge and discharge of the electrode capacitance with the electrolyte (1X phosphate-buffered saline, PBS). The nearly rectangular shape of I_bg_ at voltages above 0.3 V indicates a fully capacitive response due to the graphitic nature of our sensors. In the presence of dopamine, the total current consists of an additional component due to the redox reaction of dopamine (redox current; the bumps in the blue curve in Fig. 3(c)).

The subtraction of currents measured with and without dopamine gives the cyclic voltammogram of dopamine (Fig. 3(d)). A cyclic voltammogram contains several important details. First, the redox peak positions serve as fingerprints for the identification of target analytes. Our NG micro-sensors gave sharp and well-defined oxidation and reduction peaks to dopamine, which occurred at 380 ± 20 mV and −60 ± 20 mV. This feature of our sensors is readily useful for enhancing the identification of analytes as the oxidation and reduction peaks. Second, the distance between the two peaks (denoted as ∆E_p_) as well as the ratio of the current peak amplitudes (denoted as I_ratio_ = I_red_/I_ox_) provide qualitative insights into the kinetics of the electron transfer^8^. We measured ∆E_p_ of 440 mV and I_ratio_ of >0.8 for our NG micro-sensors. In comparison, typical carbon fiber (CF) electrodes, which are examples of fully disordered sp^2^ hybridized carbon, generally have ∆E_p_ of 600–700 mV and I_ratio_ of ∼0.65^8^ when measured using an identical FSCV waveform. Note that a smaller ∆E_p_ and a larger I_ratio_ qualitatively indicate faster electron transfer kinetics. Hence, these observations illustrate the superior electrochemical properties of our sensors. These properties arise because our miniaturized sensors are made of graphitic carbon materials, which have faster electron kinetics than those made of fully disordered sp^2^ hybridized carbon materials.

The amplitude of the redox current is another crucial aspect of a cyclic voltammogram, which quantifies the analyte concentration. This sensor characteristic depends on the total number of transferred electrons into (out of) the electrode during the oxidation (reduction) of analytes across the sensor surface (see Fig. 3(b)). A simple approach for enhancing the strength of this signal is to increase the sensor surface area. However, this approach is less desirable in sensing experiments for two main reasons. First, similar to the redox current, the amplitude of the background current also increases with the sensor surface area. As pointed out earlier, the increase of the background current negatively impacts the limit of detection of the sensor, preventing improvement in the signal-to-noise ratio of FSCV measurement of analyte concentrations. Second, increasing the sensor dimensions limits the spatial resolution of the sensor array, compromising a core benefit of sensor miniaturization. Hence, a more appropriate approach for improving the sensor performance is to engineer the material structure to achieve better S-B ratios for a constant sensor surface area.

### Correlation between structural properties of NG carbon and main sensor characteristics

As explained earlier, one viable strategy for efficient engineering of the material synthesis process is to develop phenomenological models that can relate the main sensor characteristics with quantifiable and tunable structural properties. To do so, we estimated the density of structural defects of our preferentially oriented NG carbon materials using a recent theoretical advance in the field of Raman spectroscopy^64^. Specifically, this technique provides an analytical framework for quantifying the average distance between point defects (L_D_) and the average distance between line defects (L_a_) from the Raman spectrum of graphene-based materials, including NG carbon. In brief, this technique uses the full-width at half-maximum of the G peak and the ratio of the D peak area to the G peak area for quantifying L_D_ and L_a_. From the Raman data of each NG micro-sensor, we quantified the average density of point defects (defined as n_0D_ = 1/L_D_^2^) and the average graphitic crystallite size (defined as L_a_^2^).

Figure 3(e) shows an example Raman spectrum of an NG micro-sensor. The G and D peaks are discernible. Moreover, the shape of the 2D peak indicates the turbostratic structure (i.e., rotational misalignment between the basal planes within the film) of our NG carbon material^66^. The high intensity of the 2D peak further indicates that the NG material is in stage (i) of the graphene amorphization trajectory^67,68^, where the defect-induced density of electronic states is mostly non-overlapping. All NG sensing materials used in our study had a relatively sharp G peak and noticeably strong 2D peak intensity. Hence, our NG films were far from stage (ii) of graphene amorphization trajectory, where the material is fully disordered (but not yet amorphous).

**Figure 4:**
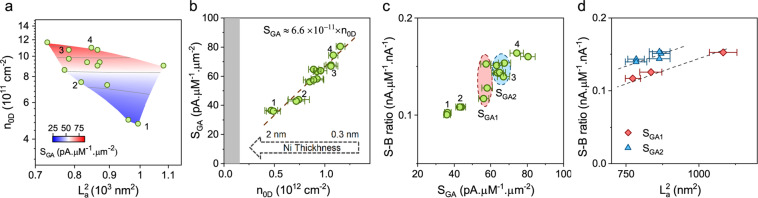
Effect of material structure on main sensor characteristics. (**a**) Contour plot of S_GA_ against n_0D_ and L_a_^2^. (**b**) The plot of S_GA_ against n_0D_, showing a linear trend. The grey shading represents the region where n_0D_ is below the detection limit of Raman. The error bars of S_GA_ are ±5% and come from the uncertainties in calculations of the sensor area including the contribution of the surface roughness. (**c**) Scatter plot of S-B ratio against S_GA_. The data show two sensor groups (denoted as S_GA1_ and S_GA2_), where sensors within each group had similar S_GA_ but different S-B ratio. (**d**) The monotonic increase of the S-B ratio with L_a_^2^ for these two sensor groups suggests the effect of L_a_^2^ on the background current. The dashed lines are guide for the eyes. The numbers next to the data points (1, 2, 3, 4) indicate the device names of four representative NG micro-sensors, which cover the entire range of measured S_GA_ and S-B ratio in our experiments. The FSCV characteristics of these sensors are shown in the Supplementary Information. The error bars of the S-B ratio are less than ±4%.

Next, we studied how these quantified structural defects shape the redox current and the background current of the sensors. Since both increase with the sensor surface area, we specifically studied how the material structure affects these two sensor characteristics normalized for unit surface area. We note that the sensors used in our study had a relatively smooth surface topography with a root-mean-square (RMS) roughness of 10 nm or less (as shown in Fig. 3(f)). Therefore, we used the geometric area (defined as the product of the sensor width and length) for estimating the sensor surface area. We evaluated the sensitivity of each sensor, with units of nA/µM, by normalizing the measured redox current to the DA concentration. Then, we calculated sensitivity per unit area (S_GA_) by normalizing the sensitivity to the sensor geometric area. As shown in the contour plot in Fig. 4(a), the size of the graphitic crystallites appears to play a negligible role in shaping S_GA_. However, S_GA_ increases with n_0D_. Interestingly, plotting S_GA_ against n_0D_ in Fig. 4(b) reveals a linear trend, where S_GA_ ≈ 6.6×10^−11^×n_0D_ with an R-squared value of 0.93.

Our results indicate the primary role of point defects in shaping the area-normalized sensitivity of the NG sensors. In particular, this linear trend suggests that each point defect acts as an individual active electrochemical site in shaping the FSCV sensitivity of dopamine by promoting adsorption and electron transfer at each site. These observations are consistent with the findings of our previous study on the FSCV sensitivity of dopamine in graphene-based electrodes^26^.

Next, we examined how increasing S_GA_ influences the S-B ratio (see Fig. 4(c)). In our calculations of the S-B ratio, we used the “intrinsic” background current that originates from the sensor material by removing the effect of the parasitic background current from the measured background current (Section S2 in Supplementary Information for details). This parasitic background current was due to the coupling between the substrate and the metal contact to the sensor^24^. The data in Fig. 4(c) show that the enhancement of S_GA_ did not result in a commensurate increase in the S-B ratio. In particular, while S_GA_ increased by almost a factor of 2.3, the S-B ratio increased by about 1.6 times. This observation may not be surprising because point defects could also increase the amplitude of the background current, reducing the overall increase of the S-B ratio. In particular, past experimental and theoretical studies have established the role of point defects in increasing the capacitance of graphene-based materials in ionic solutions^14,69–71^. The intrinsic background current in voltammetry experiments is proportional to the magnitude of this capacitance. However, we found that point defects alone are not sufficient to explain the exact variations in the S-B ratio data. A linear regression fit to the S-B ratio against the density of point defects revealed that point defects explain only ∼51% of the observed variance of the S-B ratio (Supplementary Fig. S3). This observation suggests that other factors besides point defects contribute to the background current amplitude, and hence the S-B ratio.

Figure 4(c) reveals two groups of sensors with similar S_GA_, hence a similar density of point defects, but noticeably different S-B ratios. These sensors are denoted as S_GA1_ and S_GA2_. We used this unexplained variation to investigate factors (other than the density of point defects) that influence the S-B ratio. We plotted the S-B ratio of these sensors against the average size of the graphitic crystallites, the other quantifiable parameter by Raman. The plot in Fig. 4(d) shows a monotonic increase of the S-B ratio with the average size of the graphitic crystallites. Given the similar magnitude of S_GA_ within each sensor group, we attributed the increase of the S-B ratio to the decrease of the background current and hence the sensor capacitance with increasing the crystallite size. Interestingly, this observation is in qualitative agreement with previous experimental reports. In particular, past studies show that the capacitance can range from ~2 µF.cm^−2^ for electrodes made of HOPG (which generally has micro-scale crystal domains)^14,72^ to over 200 µF.cm^−2^ for micro-supercapacitors made of reduced graphene oxide (which generally has nano-scale crystal domains)^73–75^.

To examine how the density of point defects and the size of graphitic crystallites shape the capacitance, we evaluated the area-normalized capacitance of our NG micro-sensors. We first calculated the total capacitance, which is the intrinsic background current divided by the scan rate of the FSCV waveform (i.e., 400 V/s). Then, we normalized the capacitance to the geometric area of the sensors (the same as those used for calculating S_GA_). We refer to this quantity as the apparent capacitance (C_app_).

**Figure 5:**
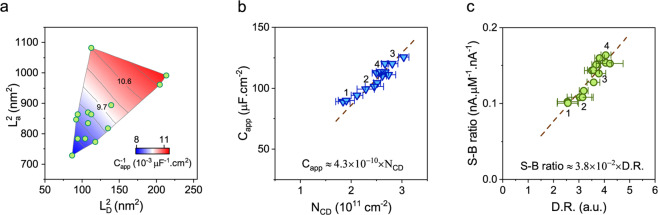
Relationship between the material structure and S-B ratio. (**a**) Contour plot of the inverse of C_app_ against L_D_^2^ and L_a_^2^. We marked two contour lines with their corresponding ${{\boldsymbol{C}}}_{{\boldsymbol{app}}}^{-1}$ values in units of 10^−3^ μF^−1^.cm^2^. (**b**) C_app_ increases in linear proportion with N_CD_, which is a function of L_D_^2^ and L_a_^2^. The error bars of C_app_ are ±5% and come from the uncertainties in calculations of the sensor area. The numbers next to the data points represent the same sensors as those in Fig. 4(c). The S-B ratio data show a linear dependence on D.R., defined as the ratio of n_0D_ to N_CD_.

C_app_ depends on the density of point defects^61–64^ (i.e., n_0D_ = 1/L_D_^2^). It also increases with the inverse of L_a_^2^ (see Fig. 4(d)). Therefore, we plotted the inverse of C_app_ against L_D_^2^ and L_a_^2^ to reveal the joint relationship among these quantities (Fig. 5(a)). Interestingly, the contour lines in this plot are nearly parallel for the entire range of our data, suggesting that a linear combination of L_D_^2^ and L_a_^2^ can best fit the inverse of C_app_. The best linear fit is given by

1 $${C}_{app}^{-1}\approx 2.3\times {10}^{9}\left(\frac{{L}_{a}^{2}}{3}+{L}_{D}^{2}\right)$$

As shown in Fig. 5(b), we found that, for the range of defects present in our sensor samples, the apparent capacitance is approximated by C_app_ ≈ 4.3×10^−10^×N_CD_. In this expression, N_CD_ is defined as (L_a_^2^/3 + L_D_^2^) ^−1^. This linear regression model had an R-squared value of 0.81, indicating that L_a_^2^ and L_D_^2^ explain 81% of the variance of C_app_. Although L_a_^2^ and L_D_^2^ explain the majority of C_app_ variance, the less than perfect R-squared value leaves room for other factors that may also affect C_app_. Surface functional groups could be one of them. However, such additional factors, if present, can only explain a small portion of the C_app_ variance. Despite its simplicity, our model provides good predictions for C_app_ and, therefore, guidelines for optimizing the material synthesis process. In particular, our capacitance model indicates that, for a given density of point defects, NG carbon micro-sensors with a larger crystallite size have smaller C_app_.

Optimization of the S-B ratio of NG sensors is consequential for improving the limit of detection. However, before investigating empirical models of S-B ratio that build on our new understanding of C_app_, it is useful to elaborate on how the S-B ratio influences the signal-to-noise ratio of NG micro-sensors. Recall that in FSCV measurements, a large background current always accompanies the redox current (i.e., the signal of interest). The amplitude of this background current in sensing measurements dictates the specifications of the readout circuitry, including the noise^48,76^. This means that, when recording the total current, sensors with the same background current amplitude have similar levels of noise. Among those, however, sensors with higher S-B ratio generate a stronger signal for a given concentration of an analyte (due to larger sensitivity), hence resulting in a higher signal-to-noise ratio and a better detection limit. This brief discussion highlights the importance of tuning the S-B ratio.

To provide quantitative guidelines for the S-B ratio optimization, we built on the models for S_GA_ and C_app_. Given the linear trends of these models, we expect that the measured S-B ratio data show a linear relationship with the ratio of n_0D_ and N_CD_, which we define as the defect ratio, D.R. = n_0D_/N_CD_. Indeed, the plot in Fig. 5(c) provides a linear model for the S-B ratio as a function of D.R. (with an R-squared value of 0.86). Our data illustrate the effectiveness of our material engineering in producing planar micro-sensors with superior S-B ratio as high as 0.17 nA.μM^−1^.nA^−1^, which is up to 4 times better than the state-of the-art single-site electrodes made of carbon fibers (e.g. see ref. ^7,8,10^). Further improvements should be attainable by maintaining a high density of point defects in the material and increasing the graphitic crystallite size.

Lastly, we remark that one possible surface feature that often exists in thin-film pyrolyzed carbon materials is nanoscale pinholes^15,77,78^, which could increase the actual surface area. However, the effect of pinholes on the actual surface area is challenging to quantify in thin-film carbon materials. Therefore, our calculations of S_GA_ and C_app_ did not account for the effect of possible nanoscale pinholes on the sensor area. Interestingly, even without adjusting the surface area for pinholes, S_GA_ and C_app_ showed a strong linear relationship against n_0D_ and N_CD_. These observations suggest that nanoscale pinholes either had a minimal effect on the actual surface area of the sensors or influenced the surface area similarly among all sensors to preserve a linear trend. On the other hand, the amplitudes of both the redox current and the background charging current generally scale similarly with the sensor area, causing the S-B ratio to be independent of the surface area. As a result, a model for predicting the S-B ratio from the material structure is robust to inaccuracies in measurements of the sensor area. The linear trend of the S-B ratio is, therefore, another strong validation of the linear trends of S_GA_ and C_app_, at least for the range of defect densities studied here.

## Discussion

The results presented in this study establish a new nano-engineering strategy for building NG electrochemical micro-sensors on dielectric-coated silicon substrates. This strategy relies on a two-pronged approach. First, we introduced a variant of the metal-induced graphitization technique that generates preferentially oriented NG carbon materials with tunable structural properties. Second, we found empirical relationships that relate the material structure (quantified by Raman) to the FSCV characteristics of the resulting micro-sensors. These models significantly simplify sensor development by reducing the number of attempts required for optimizing the synthesis process.

Our NG micro-sensors have multiple remarkable characteristics that make them an attractive choice for sensing experiments. They reproducibly generate sharp redox peaks at well-defined potentials, which is highly useful for the identification of analytes. Further, our sensors produce large redox currents for a small surface area, evident from their remarkably large S_GA_. This characteristic is highly desirable for building a dense array of micro-sensors to monitor the spatial distribution of analytes with high resolution. However, creating a functional and readily usable dense sensor array for FSCV measurements requires advances on multiple fronts, including reproducibility of sensor properties, precision of data analysis, and integrated circuits for high-precision readout of sensors. The fully capacitive characteristic of the background charging current of our NG micro-sensors provides a foundation for the necessary advance on the data analysis front by facilitating the use of data analytic techniques (see examples of some recent efforts in refs. ^79,80^). Further, the high S-B ratio of the NG micro-sensors enables the co-development of new FSCV-specific integrated circuits. These circuits are not only compact and low-power but also capable of recording both the redox and background currents with high accuracy. These striking features of our NG micro-sensors originate from their fully graphitic structure and from the ability to tune the material structure in stage (i) of the graphene amorphous trajectory. To the best of our knowledge, this study is the first to report miniaturized FSCV sensors from thin-film carbon nanomaterials that simultaneously offer these vital characteristics.

Consistency of the sensor characteristics over time is an important consideration for practical applications. For the range of defects studied here, our NG micro-sensors exhibited FSCV sensitivity to dopamine that remained stable over time during *in vitro* experiments (Supplementary Fig. S4). Our stability experiments indicate the suitability of our sensors for acute measurements of dopamine. However, an important future direction for utilizing the NG micro-sensors in the brain is to study their resistance to various sources of biofouling^81,82^. Furthermore, unlike the acute measurements, the chronic measurements of dopamine in the brain requires sensors that are stable generally for several months^83^. Hence, given the critical effect of structural defects on the sensor performance, another important future research is to study thermodynamic stability of defects in NG micro-sensors.

Finally, while the main objective of this study was to develop phenomenological models for enhancing the efficiency of the material synthesis process, our findings suggest additional research directions to be pursued in the future. Specifically, the linear trends observed for S_GA_ and C_app_ suggest fundamental mechanisms by which point defects and crystallite size in NG carbon materials affect these electrochemical properties. However, the extent of details on the material structure provided by Raman spectroscopy is not sufficient to fully unravel the underlying mechanisms. Additional studies that accurately quantify the sub-types of defects in NG carbon materials and explore their relationship with FSCV sensor characteristics will be essential to discover the fundamental working mechanisms of the NG micro-sensors. These studies could also provide critical insights into the factors that shape the slope of the observed linear trends. These details will be highly valuable for refining the optimization process of the NG micro-sensors.

## Methods

### Material Synthesis and device fabrication

The material synthesis builds on our previous study^26^. The starting material was SU-8 2002 resist spun cast onto SiO_2_-covered silicon substrates at 4000 rpm. The carbonization step was performed at 450 °C and in an 80/20 mixture of Ar/H_2_ (a flow rate of 500 sccm) for one hour. Nickel deposition was performed in an UHV system at a base pressure of ∼1×10^−10^ mbar and at a deposition rate of about 0.2 nm per minute. Graphitization step was performed at 1100 °C for 30 min at atmospheric pressure in an 80/20 mixture of Ar/H_2_ gas carrier (flow rate of 50 sccm). All resist patterning steps were performed using EBL. The metal contacts to the NG carbon islands were made through e-beam evaporation of 10 nm Cr/50 nm Au at a high vacuum of about 10^−7^ Torr.

### Raman Measurements

To quantify the structural defects of the NG sensors, we followed a procedure similar to ref. ^26^. In brief, Raman spatial maps were first obtained using the Horiba Xplora micro-Raman system with a 532 nm laser. The Raman spectra were then fitted to evaluate the full-width at half-maximum (FWHM) of the G peak and the area ratio of the G and D peaks. Lastly, these curve fitting results were used to extract the average density of point defects (n_0D_) and the average crystallite size (L_a_^2^) according to the theoretical framework described in ref. ^64^.

### Electrochemical Measurements

The current signal was measured using a low-noise current amplifier (SR570, Stanford Research Systems) and subsequently digitized using a data acquisition instrument (NI USB-6353X series, National Instruments). A custom-made LabVIEW control interface was used to operate these instruments.

## Supplementary information

Supplementary information.
