# Supplementary material for: Nano-engineering the material structure of preferentially oriented nano-graphitic carbon for making high-performance electrochemical micro-sensors
Source: Sci Rep. 2020 Jun 10;10:9444. doi: 10.1038/s41598-020-66408-9 (PMC7286892; doi:10.1038/s41598-020-66408-9)
Supplement: Supplementary file 1 — Supplementary information. [file 41598_2020_66408_MOESM1_ESM.pdf]

Supplementary Information

Nano-engineering the material structure of preferentially oriented nano-graphitic carbon for making high-performance electrochemical micro-sensors

Edoardo Cuniberto,[1Ɨ] Abdullah Alharbi,[1,2Ɨ] Ting Wu,[3] Zhujun Huang,[1] Kasra Sardashti, Kae-Dyi You,[1] Kim K isslinger,[5] Takashi Taniguchi,[6] Kenji Watanabe,[6] Roozbeh Kiani,[3,7] Davood Shahrjerdi [1,4*]

[1] Electrical and Computer Engineering, New York University, Brooklyn, NY 11201,

[2] National Center for Nanotechnology and Semiconductors, KACST, Riyadh, Saudi Arabia 11442,

[3] Center for Neural Science, New York University, New York, NY, 10003,

[4] Center for Quantum Phenomena, Physics Department, New York University, New York, NY 10003,

[5] Center for Functional Nanomaterials, Brookhaven National Laboratory, Upton, NY 11973,

[6] National Institute of Materials Science, 1-1 Namiki Tsukuba, Ibaraki 305-0044, Japan,

[7] Department of Psychology, New York University, New York, NY 10003

[*] E-mail: davood@nyu.edu. [Ɨ] These authors contributed equally to this work.

S1. FSCV characterization of NG sensors

Figure S1 shows the FSCV characteristic of four representative NG sensors with different structural properties, i.e., average density of point defects and average crystallite size. These sensors were denoted as devices 1-4 in the main text (in Figures 4 and 5). Figure S1(a) shows the redox current of these sensors normalized to the dopamine concentration and to their corresponding geometric area, denoted as $I_{normalized}$. Figure S1(b) shows the corresponding background current of these sensors. The data in Figure S1 clearly show that the output characteristics of our NG sensors differ from each other in terms of the magnitude of the current signals, while their overall shapes are consistent. This latter observation suggests that the underlying mechanisms that shape the redox current and the background current are consistent among our NG sensors. This consistency in the overall characteristics of our NG sensors allowed us to build the phenomenological models around the density of point defects and the size of the graphitic crystallites.

As can be seen in Figure S1(b), the background current of device 4 shows a sudden increase at voltages above 0.8 V. We generally observed this behavior for sensors fabricated on thin oxide films (in this case 90 nm thick SiO_2). This observation points to a potential reliability issue when building sensors on thin dielectrics. However, the use of thicker oxides fully resolved this issue. As we describe in the next section, the use of thick oxides reduces the parasitic current, I_{par}, which is also an undesirable feature since it adds to the amplitude of the background current. The origin of this parasitic current is the capacitive coupling of the metal contacts with the silicon substrate.

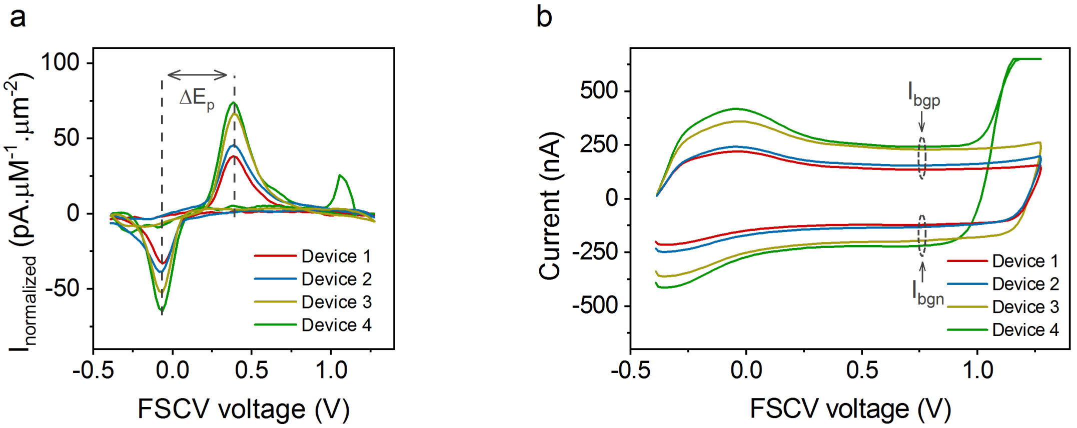

Figure S1. FSCV characteristics. (a) Normalized cyclic voltammograms and (b) the measured background currents of four representative NG sensors with different density of point defects and crystallite size. The redox characteristics of these sensors in panel (a) show the consistency in the positions of oxidation and reduction peaks, giving a ΔE_p of 440 ± 20 mV. The data in panel (b) show the consistent shapes of the background current of our sensors. For calculations of the intrinsic background current, we used the data in the regions denoted as I_{bgp} and I_{bgn}. Note that the measured background currents in panel (b) include the parasitic current, I_{par}. The parasitic current for device 1, 2, 3, and 4 were 18 nA, 15 nA, 52 nA, and 52 nA, respectively. The extraction of the I_{par} is explained in section S2.

S2. Extraction of the intrinsic background current

To study the effect of the material structure on the S-B ratio, we must consider only the background current due to the sensor capacitance, which we refer to as the intrinsic background current. To do so, we first evaluated the background current as $I_{bg} = (I_{bgp} - I_{bgn})/2$, which is the average of the positive and negative background currents at the same potential in the flat region of the measured current-voltage curve, as shown in Figure S1(b). We then subtracted the contribution of the measured parasitic current, as we explain next, from I_{bg}. We use the intrinsic background current of each sensor for evaluating its S-B ratio and capacitance.

The capacitive coupling between the metal leads and the substrate produces a parasitic current. To accurately account for this parasitic current, we fabricated test structures on every sample sensor. Figure S2(a) shows the optical image of an example test structure. The test structures were identical to the NG sensors, except that the NG film was covered completely with the SU-8 protection layer. We directly measured the parasitic current of each sensor sample by applying the FSCV waveform (the same as what was used for dopamine measurements) to their corresponding test structures. Figure S1(b) shows the current-voltage characteristic of an example test structure. In our experiments, we noticed that the magnitude of the parasitic current depends on the thickness of the SiO_2 dielectric and the dimensions of the metal lead. Table S1 gives the summary of the measured parasitic current amplitudes for a few different test structures.

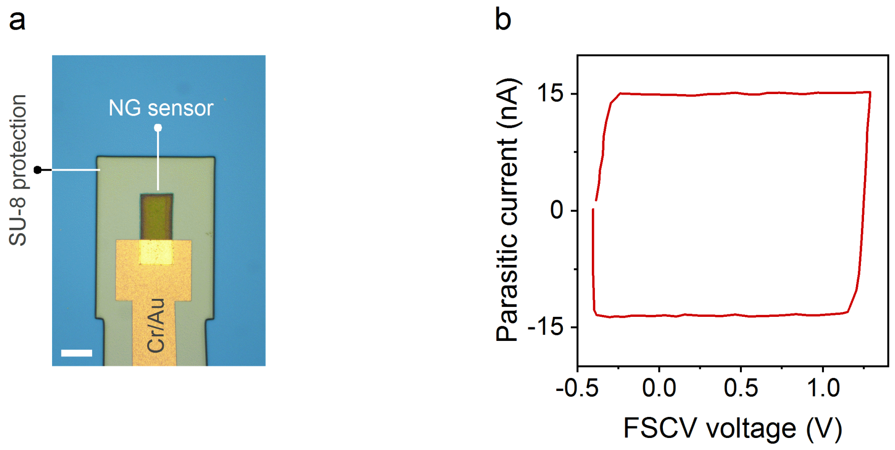

Figure S2. Extraction of the parasitic current. (a) Optical image showing a test structure produced by fully covering the NG film with the SU-8 protection layer. The scale bar is 20 μm. (b) An example of the parasitic current measured for a test structure with a 40 μm wide metal line, fabricated on a 490 nm thick SiO_2. The measurement was performed at 400 V/s scan rate and a repetition frequency of 10 Hz.

Table 1: Summary of the measured parasitic currents for a few test structures.

SiO_2 thickness (nm)	Metal line width (µm)	Parasitic current (nA) at 400 V/s
90	40	52
300	5	10
330	40	18
490	40	15

S3. Linear regression fit of S-B ratio against point defects

Figure S3 shows the scatter plot of the S-B ratio data against the density of point defects (n_{0D}) for our NG sensors, showing that the S-B ratio increases with the density of the point defects. A linear fit to the data gave an R-squared value of 0.51, indicating that point defects alone are not sufficient for predicting the S-B ratio of our sensors. Indeed, we found that the size of the graphitic crystallites is yet another factor that contributes to shaping the amplitude of the background current and hence the S-B ratio.

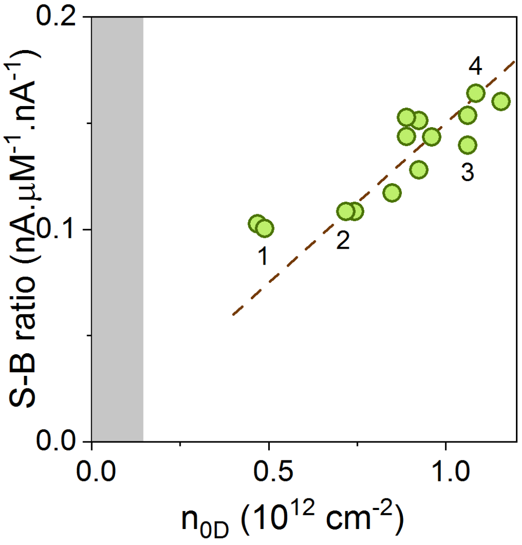

Figure S3. Linear regression of S-B *vs* n_{0D}. Scatter plot of the S-B ratio against n_{0D}. The linear regression fit (dashed line) has an R-squared of 0.51. The shading represents the region where the point defect density is below the detection limit of Raman.

S4. Electrochemical stability of NG micro-sensors

We studied the electrochemical stability of our NG micro-sensors to dopamine by performing FSCV measurements for a prolonged period of time. The data in Fig. S4 show the measurement results for an NG micro-sensor. In this experiment, the FSCV waveform was applied continuously to the sensor for up to 90 min. Dopamine was introduced at different times during the experiment, denoted in the plot. The cyclic voltammogram corresponding to each dopamine injection was

obtained. The data in Fig. S4 show the consistency of the cyclic voltammograms measured over time, confirming the stability of the NG micro-sensors for acute measurements of dopamine.

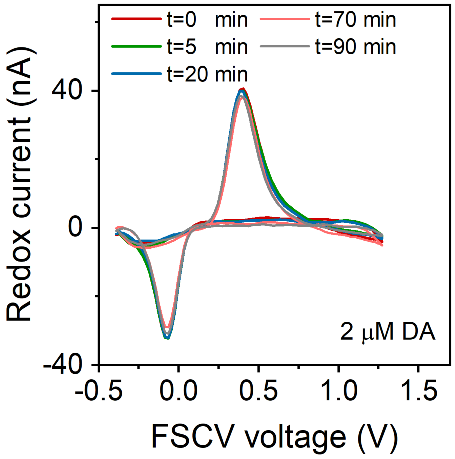

Figure S4. Electrochemical stability of NG micro-sensors. Cyclic voltammograms of dopamine measured at different times using an NG micro-sensor. The FSCV waveform was applied to the sensor continuously for 90 min, while dopamine was injected at specific times denoted in the plot.
